# Linearization of ancestral multichromosomal genomes

**DOI:** 10.1186/1471-2105-13-S19-S11

**Published:** 2012-12-19

**Authors:** Ján Maňuch, Murray Patterson, Roland Wittler, Cedric Chauve, Eric Tannier

**Affiliations:** 1 Department of Mathematics, Simon Fraser University, Burnaby BC, V5A1S6, Canada; 2 Department of Computer Science, University of British Columbia, Vancouver BC, V6T1Z4, Canada; 3 INRIA Rhône-Alpes, 655 avenue de I'Europe, F-38344 Montbonnot, France; 4 Laboratoire de Biométrie et Biologie Évolutive, CNRS and Université de Lyon 1, 43 boulevard du 11 novembre 1918, F-69622 Villeurbanne, France; 5 Genome Informatics, Faculty of Technology and Institute for Bioinformatics, Center for Biotechnology (CeBiTec), Bielefeld University, 33594 Bielefeld, Germany

## Abstract

**Background:**

Recovering the structure of ancestral genomes can be formalized in terms of properties of binary matrices such as the Consecutive-Ones Property (C1P). The *Linearization Problem *asks to extract, from a given binary matrix, a maximum weight subset of rows that satisfies such a property. This problem is in general intractable, and in particular if the ancestral genome is expected to contain only linear chromosomes or a unique circular chromosome. In the present work, we consider a relaxation of this problem, which allows ancestral genomes that can contain several chromosomes, each either linear or circular.

**Result:**

We show that, when restricted to binary matrices of degree two, which correspond to adjacencies, the genomic characters used in most ancestral genome reconstruction methods, this relaxed version of the Linearization Problem is polynomially solvable using a reduction to a matching problem. This result holds in the more general case where columns have bounded multiplicity, which models possibly duplicated ancestral genes. We also prove that for matrices with rows of degrees 2 and 3, without multiplicity and without weights on the rows, the problem is NP-complete, thus tracing sharp tractability boundaries.

**Conclusion:**

As it happened for the breakpoint median problem, also used in ancestral genome reconstruction, relaxing the definition of a genome turns an intractable problem into a tractable one. The relaxation is adapted to some biological contexts, such as bacterial genomes with several replicons, possibly partially assembled. Algorithms can also be used as heuristics for hard variants. More generally, this work opens a way to better understand linearization results for ancestral genome structure inference.

## Introduction

Genomes, meant as the linear organization of genes along chromosomes, have been successively modelled by several mathematical objects. Sturtevant and Tan [1] first introduced permutations to study the evolution of genome structure. Starting in the 1980's [2], a large body of work focused on the mathematical and algorithmic properties of such models, including linear and circular genomes [3]. Multichromosomal linear genomes have been defined as generalizations of permutations: they are permutations cut in several pieces [4]. In this framework, hardness results of algorithmic complexity were ubiquitous as soon as three genomes were compared [5,6]. Even worse, if strings were used to model duplications and heterogeneous gene content, then even the basic problem of comparing two genomes proved to be hard [7].

In order to scale up and handle the dozens of available genomes, another model was needed. Bergeron, Mixtacki and Stoye [8] proposed to use a graph matching between gene extremities to define a genome. It simplified the presentation of the Double-Cut and Join (DCJ) theory [9] at the expense of relaxing the model of chromosomal structure as genomes could contain both linear and circular chromosomes. This can be seen as an unrealistic relaxation, as genomes are mostly either linear multichromosomal (eukaryotic nuclear genomes) or circular unichromosomal (bacterial or organelle genomes). But eukaryotes with organelles, or prokaryotes with several replicons, which have not yet been handled explicitly by a formal comparative genomics approach, arguably fit such a model. An unexpected consequence of this relaxation is that the comparison of three genomes with the breakpoint distance proved to be tractable, as an exact optimal median can be computed by solving a maximum weight perfect matching problem [10]. Moreover, the small parsimony problem, *i.e*., reconstructing the minimum number of evolutionary events along a given phylogeny, can be solved for any number of genomes for the Single-Cut and Join (SCJ) distance by Fitch's parsimony algorithm on binary characters [11]. This opened the way to scalable methods at the level of large multispecies datasets.

An additional relaxation consists in allowing any graph, and not only a matching, to model genomes. Ancestral genome reconstruction methods often first compute sets of ancestral adjacencies (neighborhood relations between two genes) [12-14], intervals (neighborhood relations between an arbitrary number of genes) [15-18], which result in non-linear structures. This, while also unrealistic at a first glance, allows computational breakthroughs, like incorporating duplications and heterogeneous gene content in the framework [19,20] with polynomial exact methods. Beyond the significant computational speedup, nonlinear genomes may help to understand the amount of error in the data [20].

Nevertheless, biological applications in general require linear genomes, which raises the question of *linearizing *a collection of adjacencies or intervals. The *Linearization Problem *is, given a set of weighted intervals (the weight indicates a confidence value based on phylogenetic conservation of intervals), to find a maximum weight subset which is compatible with a linear structure.

According to the definition of a linear structure, this can be described by some variant of the Consecutive-Ones property (C1P) of binary matrices [10,15,17]: A binary matrix has the C1P if its columns can be ordered such that in each row, there is no 0 entry between two 1 entries. Here, each column is a gene or a gene extremity and each row is an interval. Adjacencies are a particular case of intervals of size two: In that case, the matrix, which has degree 2, can be identified with a graph (vertices are columns and edges are rows). In the case of adjacencies, the Linearization Problem translates to the Maximum Weight Vertex-Disjoint Path Cover Problem, so it is NP-complete. A variant handles genomes with a single circular chromosome: A binary matrix has the circular C1P (Ci1P) if its columns can be ordered such that in each row, either there is no 0 entry between two 1 entries, or there is no 1 entry between two 0 entries. For adjacencies, the Linearization Problem contains the Maximum Weight Hamiltonian Cycle Problem, so it is also NP-complete.

To the best of our knowledge, there is currently no tractability result known for the Linearization Problem. Currently all methods [12-14,19] rely on heuristic or external Traveling Salesman Problem solvers, or branch and bound techniques [10,15-18]. Moreover, none of the previously published methods is able to infer multichromosomal genomes, possibly with circular chromosomes, which is the natural model for bacterial genomes with plasmids.

In the present paper, we prove that the Linearization Problem for weighted adjacencies, when ancestral genomes can have several circular and linear chromosomes, is tractable. We prove this in a more general case, where multiple copies of columns are allowed. Here, instead of a permutation of the columns, one asks for a sequence on the alphabet of columns, containing at most *m*(*c*) occurrences of a column *c*. In the context of genome reconstruction, this allows to model genes with multiple copies in an ancestral genome [21] or to include telomere markers [22].

We show that this corresponds to finding a maximum weight *f*-matching, which, in turn, is reducible to finding a maximum weight matching. Also, following the complexity pattern already observed with the model of the C1P with multiplicities [21], we further show that the Linearization Problem for matrices with rows of degrees 2 and 3 is NP-complete, even if all rows have the same weight and multiplicity one. We discuss the possibilities that our tractability result opens for ancestral genome reconstruction.

## Results

A few definitions are needed to prove the two main results of this paper: (1) a polynomial algorithm for the linearization of degree 2 matrices with columns with multiplicity and weighted rows; and (2) an NP-completeness proof for the linearization of matrices with rows of degrees 2 and 3, even if all multiplicities and row weights are equal to one.

The *degree *of a row of a *binary *matrix *M *(over {0, 1}) is the number of 1 entries in that row. The degree of *M *is the maximum degree over all its rows. In genomics, the columns of *M *are the *genes*, and its rows are the *intervals *of genes. If a row has degree 2, the interval is called an *adjacency*. A degree 2 matrix *M *can be identified with a graph, whose vertices are the columns of *M*, and edges are the adjacencies. We suppose that all rows are different (and in consequence the graph is simple: it has no multi-edges).

A binary matrix (or submatrix) *M *has the *Consecutive Ones Property *(C1P) if its columns can be ordered such that in each row, the 1 entries are consecutive (there is no 0 entry between two 1 entries). If *M *has degree 2, it has the C1P if and only if the corresponding graph is a collection of vertex-disjoint paths. A matrix (or submatrix) *M *has the *Circular Ones Property *(Ci1P) if its columns can be ordered such that in each row, either the 1 entries are consecutive (there is no 0 entry between two 1 entries), or the 0 entries are consecutive (there is no 1 entry between two 0 entries); in other words the 1 entries are consecutive when the order of columns is viewed as a circle. If *M *has degree 2, it has the Ci1P if and only if the graph is a cycle or a collection of vertex-disjoint paths.

Given maximum copy numbers *m*(*c*) for each column *c*, *M *satisfies the *Consecutive Ones Property with multiplicities *(mC1P), if there is a sequence *S *of columns, containing at most *m*(*c*) occurrences of column *c*, and for each row *r*, the columns containing a 1 in *r *appear consecutively in *S*. The mCi1P is defined analogously.

The *MAX-ROW-C1P *problem takes a binary matrix with weighted rows as input and asks for a subset of rows of maximum cumulative weight with the C1P. For graphs it is equivalent to the Maximum Weight Vertex-Disjoint Path Cover Problem and thus the *MAX-ROW-C1P *is NP-complete [23]. The *MAX-ROW-Ci1P *problem takes a matrix with weighted rows as input and asks for a subset of rows of maximum cumulative weight with the Ci1P. For graphs it can solve the Traveling Salesman Problem and thus the *MAX-ROW-Ci1P *is NP-complete [23].

These two problems are classical and have been defined independently from comparative genomics, but model well the linearization of genomes with linear chromosomes, or a single circular chromosome, respectively. But the general case would better be modelled by the following. A matrix is *component*-*mCi1P *if there is a collection of cyclic sequences of columns that satisfy the following two conditions: (i) for each row *r*, the columns containing a 1 in *r *appear consecutively in at least one of the cyclic sequences; and (ii) the total number of occurrences of each column *c *in all cyclic sequences is at least one and at most *m*(*c*). In the particular case where *m*(*c*) = 1 for every column *c*, a matrix is *component-mCi1P *if its columns can be partitioned such that a row has 1s only in one part and each part is Ci1P. Here chromosomes are sequences, which mean possible ancestral gene orders. It is then the matter of solving the following problem.

### MAX-ROW-component-mCi1P

**Input**. A matrix with maximum copy numbers assigned to all columns and weighted rows;

**Output**. A subset of rows of maximum cumulative weight such that the obtained submatrix is *component-mCi1P*.

Note that it is equivalent if some sequences are not required to be circular, so it handles well the case where both circular and linear chromosomes are allowed. It is a relaxation of the previous problems, so the NP-hardness does not follow from them. And in fact, the problem for degree 2 matrices (adjacencies) happens to be polynomial, as we now show in the next subsection.

### A solution for matrices of degree two with weighted rows and multiplicites

For a degree 2 matrix *M*, let *G_M _*be the corresponding graph with a node for each column and a weighted edge for each (weighted) row. Let *m *: *V*(*G_M_*) → ℕ be the function specifying the maximum copy number of each column, *i.e.*, the multiplicity limit for each vertex of *G_M_*. We say that matrix *M *(resp., the corresponding graph *G_M_*) is component-mCi1P for *m *if there exists a collection of cyclic walks (resp., corresponding cyclic sequences) that satisfies the following two conditions: (i) *G_M _*is a subgraph of the union of cyclic walks; and (ii) the total number of occurrences of each vertex *v *in all cyclic walks is at most *m*(*v*).

A 2*m*-*matching *of a graph *G *is a spanning subgraph of *G *such that the degree of each vertex *v *∈ *V*(*G*) is at most 2*m*(*v*). The following lemma shows the correspondence between spanning subgraphs of *G *that are component-mCi1P for *m *and 2*m*-matchings of *G*.

**Lemma 1 ***A spanning subgraph of a graph G is component-mCi1P for m if and only if it is a *2*m*-*matching of G*.

We give a sketch of the proof. For more details, we refer the reader to [21], where a similar proof was given.

*Proof*. First, assume a spanning subgraph *G*' of *G *is component-mCi1P for *m*. Then there is a collection of cyclic walks satisfying conditions (i) and (ii). Since each vertex *v *appears at most *m*(*v*) times in these cyclic walks and each occurrence has only two neighbors, the degree of *v *in *G*' is at most 2*m*(*v*). Hence, *G*' is a 2*m*-matching of *G*.

Conversely, assume *G*' is a 2*m*-matching of *G*. If deg_*G*' _(*v*) < 2*m*(*v*) for some *v *∈ *V*(*G*'), then we add a new vertex *v*_0 _and for each *v *such that deg_*G*' _(*v*) < 2*m*(*v*), we add a new edge {*v*_0_, *v*} with multiplicity 2*m*(*v*) − deg_*G*' _(*v*) to *G*'. Since now every vertex of *G*' has even degree, each component *C *of *G*' is Eulerian, *i.e.*, there is a cyclic walk which contains all edges of *C*, and each *v *∈ *V*(*C*) appears exactly *m*(*v*) times in the walk. If *C *does not contain *v*_0 _then this cyclic walk satisfies conditions (i) and (ii) for vertices in *V*(*C*). If *C *contains *v*_0_, then after omitting all occurrences of *v*_0 _we obtain a cyclic walk satisfying conditions (i) and (ii) for vertices in *V*(*C*). Hence, *G*' is component-mCi1P for *m*. QED

It follows that solutions to the MAX-ROW-component-mCi1P for matrix *M *and *m *correspond to maximum weight 2*m*-matchings of *G_M_*. Next, we give an algorithm for finding a maximum weight *f*-matching of *G *with running time (*O*((|*V*(*G*)| + |*E*(*G*)|)^3/2^)), where *f *: *V*(*G*) → ℕ. We will use a more general form of Tutte's reduction for reducing the maximum weight *f*-matching problem to the maximum weight matching problem similar to the ones presented in [24,25].

**Figure 1:**
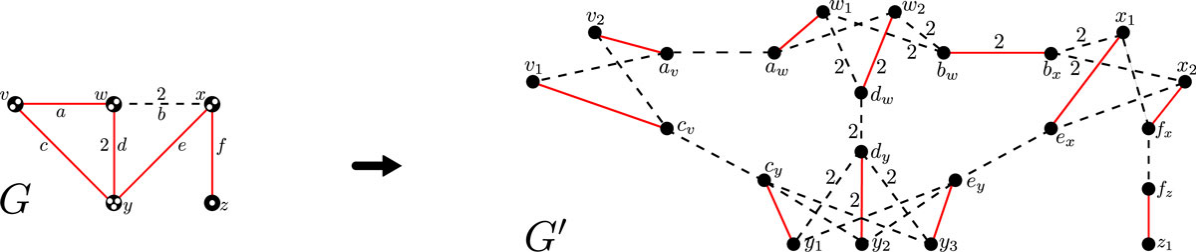
**Reduction used to transform the maximum weight *f*-matching problem to the maximum weight matching problem**. Edge weights are all one, unless otherwise indicated, and *f *is given by the white dots inside the nodes. The total edge weight in *G *is 8. The solid edges show a maximum weight *f*-matching in *G *(*w *= 6), and a corresponding maximum weight matching in *G*' (of weight 6 + 8 = 14).

Given an edge weighted graph *G *and function *f*, construct *G*' in the following way: For all *x *in *V*(*G*), let *x*_1_, *x*_2_, ..., *x*_*f*(*x*) _be in *V*(*G*'); and for all *e *= {*x*, *y*}in *E*(*G*), let *e_x _*and *e_y _*be in *V*(*G*'). Now, for all *e *= {*x*, *y*} in *E*(*G*), let {*x*_1_, *e_x_*}, ..., {*x*_*f*(*x*)_, *e_x_*}, {*e_x_*, *e_y_*}, {*y*_1_, *e_y_*}, ...,{*y*_*f*(*y*)_, *e_y_*} be edges of *G*', and all these edges have weight *w*(*e*). This reduction is illustrated in Figure 1.

**Property 1 ***There is an f*-*matching in G with weight w if and only if there is a matching in G*' *with weight w *+ *W*, *where *$W=\sum_{e\in E\left(G\right)}w\left(e\right)$.

An unweighted version of this property was shown in [25]. The weighted version can be shown in the same way, and hence, we omit the proof.

Since a maximum weight matching can be found in time $O\left(\sqrt{\left|V\left(G^{\prime }\right)\right|}\;\cdot \;|E\left(G^{\prime }\right)|\right)$[26], we have polynomial *O*((|*V*(*G*)| + |*E*(*G*)|)^3/2^) algorithms for the maximum weight *f*-matching problem and for the MAX-ROW-component-mCi1P problem with multiplicities on matrices of degree 2.

### Intractability for matrices of degree larger than two

The tractability does not generalize to matrices, that is, the MAX-ROW-component-Ci1P is already NP-complete for unweighted matrices with rows of degrees 2 and 3. Note that the result for unweighted matrices implies NP-completeness also for the cases when rows are weighted and/or columns have multiplicities.

We will first show that the following hypergraph covering problem is NP-complete. Here we say that a hypergraph *H *= (*V*, *E*) is *2*, *3*-*uniform *when all of its hyperedges are either *2*-*edges *or *3*-*edges*, that is, hyperedges that contain exactly two or three vertices. We will also denote 2,3-uniform hypergraphs *H *= (*V*, *E*) as *H *= (*V*, *E*_2_, *E*_3_), where *E*_2 _(resp., *E*_3_) is its set of 2-edges (resp., 3-edges). We denote the *power set *of a set *S *with P(S) (also known as 2*^S^*).

**Definition 1 ***A *graph covering *of a 2*, *3*-*uniform hypergraph H *= (*V*, *E*_2_, *E*_3_) *is a graph G *= (*V*, *E*') *such that there exist a map *$c:E_{2}\cup E_{3}\to P\left(E^{\prime }\right)$, *satisfying the following for every h *∈ *E*_2 _∪ *E*_3_*:*

*(a) for every h *∈ *E*, *and for every e *∈ **c**(*h*), *e *⊆ *h*;

*(b) *|**c**(*h*)| = 1 *if h *∈ *E*_2 _*and *|**c**(*h*)| = 2 *if h *∈ *E*_3_; *and*

*(c) *$\cup_{h\in E_{2}\cup E_{3}}$**c**(*h*) = *E*'.

*Here, we say that each set of edges ***c**(*h*) covers *the hyperedge h*.

Informally, a graph covering of a 2,3-uniform hypergraph is a graph constructed by picking an edge from each 2-edge, and a pair of edges from each 3-edge.

**Problem 1 (The 2,3-Uniform Hypergraph Covering by Cycles and Paths by Edge Removal Problem (23UCR Problem)) ***Given a 2, 3-uniform hypergraph H = *(*V, E*) *and an integer k, is there a graph covering of H that consists of a collection of disjoint cycles and paths after removing at most k hyperedges from E?*

Here we will show that Problem 1, the 23UCR Problem, is NP-complete. Later in this section we will show that this implies that the MAX-ROW-component-Ci1P Problem is NP-complete for matrices with rows of degrees 2 and 3. First, we must define the following NP-complete version of 3SAT, which we will use to show NP-completeness of Problem 1.

**Problem 2 (The 3SAT(2,3) Problem) ***Given a CNF formula *ϕ *with the following three properties, is *ϕ *satisfiable?*

*(a) Formula *ϕ *has only 2-clauses and 3-clauses*.

*(b) Each variable x of *ϕ *has exactly two positive occurrences and one negative occurrence in the clauses*.

*(c) Exactly one positive occurrence of x appears in the 3-clauses*, *while the other two occurrences appear in the 2-clauses*.

We show that this version of 3SAT is NP-complete using a very similar proof to the one in [27], by reduction from 3SAT.

**Theorem 1 ***The 3SAT(2,3) Problem is NP-complete*.

*Proof*. Clearly, the problem is in NP. We now show that it is NP-hard by reduction from 3SAT by transforming a given formula ϕ  that is an instance of 3SAT to a formula $\phi^{\prime }$ that is an instance of 3SAT(2,3) that is satisfiable if and only if ϕ  is satisfiable. For each variable *x *of ϕ  that has *k *occurrences, we first replace its *k *occurrences with *x*^1^, *x*^2^,..., *x^k^*, *i.e.*, replace the *i*-th occurrence of *x *(as literal *x *or ¬*x*) with *x^i^*. We then add the following 2-clauses: ${\bar{x}}^{i}\Rightarrow {\bar{x}}^{i+1}\left(i.e.,\neg {\bar{x}}^{i}\vee {\bar{x}}^{i+1}\right)$ for *i *= 1, ..., *k *- 1 and also ${\bar{x}}^{k}\Rightarrow {\bar{x}}^{1}$, where for each *i*,

$${\tilde{x}}^{i}=\{\begin{array}{ll} x^{i} & \mathrm{if}\;\mathrm{the}\;i-\mathrm{th}\mathrm{occurrence}\;\mathrm{of}\;\mathrm{variable}\;x\;\mathrm{is}\;\mathrm{positive},\;\mathrm{and}\\ \neg x^{i} & \mathrm{otherwise}. \end{array}$$

This "cycle" of implications (2-clauses) on *x*^1^,..., *x^k^*, ensures that for any truth assignment to the variables of $\phi^{\prime }$, the values of ${\bar{x}}^{1},\ldots ,{\bar{x}}^{k}$ are either all set to *true *or all set to *false*. In the first case, the *x^i^*'s corresponding to the positive occurrences of *x*, are set to *true *and the *x^i^*'s corresponding to the negated occurrence of *x*, are set to *false*. In the second case, the situation is reversed. Hence, any satisfying truth assignment to the variables of $\phi^{\prime }$ can be translated into a satisfying truth assignment to the variables of ϕ , and vice versa, *i.e.*, $\phi^{\prime }$ is satisfiable if and only if ϕ  is satisfiable. Since it is easy to verify that this transformation can be done in polynomial time, and that $\phi^{\prime }$ is indeed an instance of 3SAT(2,3), it follows that the 3SAT(2,3) Problem is NP-complete. QED

We now show that the 23UCR Problem is NP-complete by reduction from 3SAT(2,3).

**Theorem 2 ***The 23UCR Problem is NP-complete*.

*Proof*. Clearly, the problem is in NP. We will show that it is also NP-hard by reduction from 3SAT(2,3).

Given a 3SAT(2,3) formula ϕ  with variables *X *= {*x*_1_, ..., *x_n_*} and set $C_{\text{2}}=\left\{c_{\text{1}},\ldots ,c_{m_{2}}\right\}$ of 2-clauses (resp., set $C_{\text{3}}=\left\{c_{\text{1}},\ldots ,{c_{m}}_{{}_{\text{3}}}\right\}$ of 3-clauses), we construct a 2,3-uniform hypergraph *H_ϕ _*= (*V*, *E*). Hypergraph *H_ϕ _*is composed of variable gadgets and clause gadgets which contains, among other vertices, a vertex for each literal of ϕ  (what we will refer to as literal vertices: there are 3*n *= 2*m*_2 _+ 3*m*_3 _such vertices). The design of $H_{\phi }$ is such that there is a graph covering *G *of $H_{\phi }$ that consists of a collection of disjoint cycles and paths after removing at most *m*_2 _+ *n *edges from *E *if and only if ϕ  is satisfiable. For this proof, we call such a *G *a *valid *covering of *H*. Note that a valid covering does not contain any vertex of degree 3 or more.

**Figure 2:**
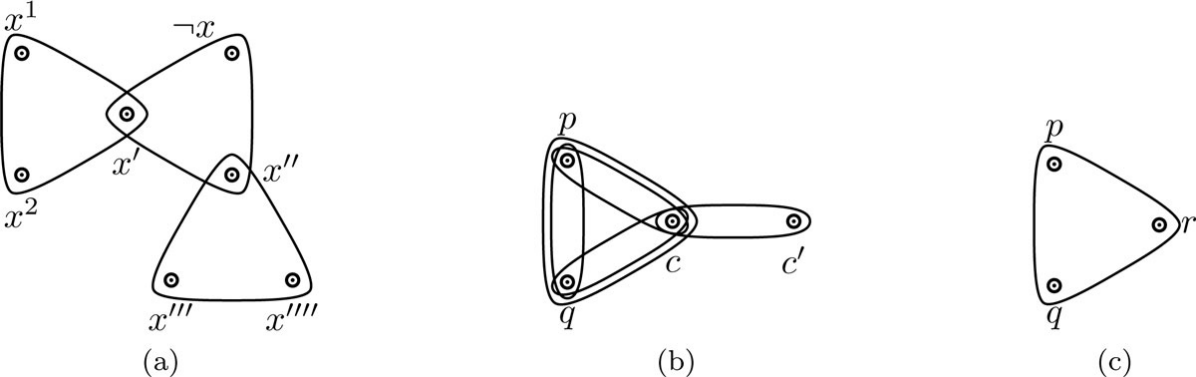
**(a) The variable gadget for variable *x *with literal vertices *x*^1^, *x*^2 ^and ¬*x*, as well as the four auxiliary vertices *x*', *x*'', *x*''', *x*'''' that do not appear in any other hyperedge of $H_{\phi }$.** (b) 2-clause gadget with literal vertices *p *and *q*, as well as the two auxiliary vertices *c *and *c*' that do not appear in any other hyperedge of *H_ϕ_*. (c) 3-clause gadget with literal vertices *p*, *q *and *r*.

Figure 2a depicts the variable gadget for variable *x *∈ *X *with its two positive occurrences, labeled as *x*^1 ^and *x*^2^, and its one negative occurrence ¬*x *in the clauses. We call the 3-edge {*x*'', *x*''', *x*'''' } the *auxiliary *hyperedge, while the other two, {*x*^1^, *x*^2^, *x*'} and {¬*x*, *x*', *x*''}, are called the *main *hyperedges of the variable gadget.

Figure 2b (resp., 2c) depicts the clause gadget for the 2-clause containing literals *p*, *q *(resp., and also *r *for a 3-clause). For the 2-clause gadget, we call the 2-edge {*c*, *c*'} the *auxiliary *hyperedge. We will refer to literals of *ϕ *and the literal vertices of the gadgets of $H_{\phi }$ interchangeably when the context is clear. We have the following claim.

**Lemma 2 ***Formula *ϕ *has a satisfying assignment if and only if *$H_{\phi }$*has a valid covering*.

*Proof*. "⇒" We first show that a satisfying assignment of ϕ  can be used to construct a valid covering of $H_{\phi }$.

**Figure 3:**
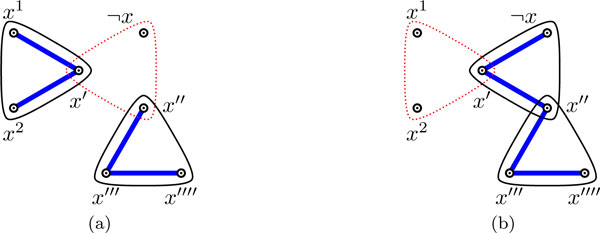
**Two coverings of the variable gadget for *x*, when *x *is set to: (a) *false*, or (b) *true *in the assignment**.

For the variable gadget corresponding to *x *∈ *X*, we first remove the main hyperedge that contains the literal(s) that is satisfied in the assignment, and then cover the remaining two edges as depicted in Figure 3: Figure 3a (resp., 3b) depicts how to cover the clause gadget when *x *is *false *(resp., *true*) in the assignment. In this figure (as in all remaining figures of this paper), hyperedges drawn with dashed lines are removed, while the straight lines are edges picked in the covering.

**Figure 4:**
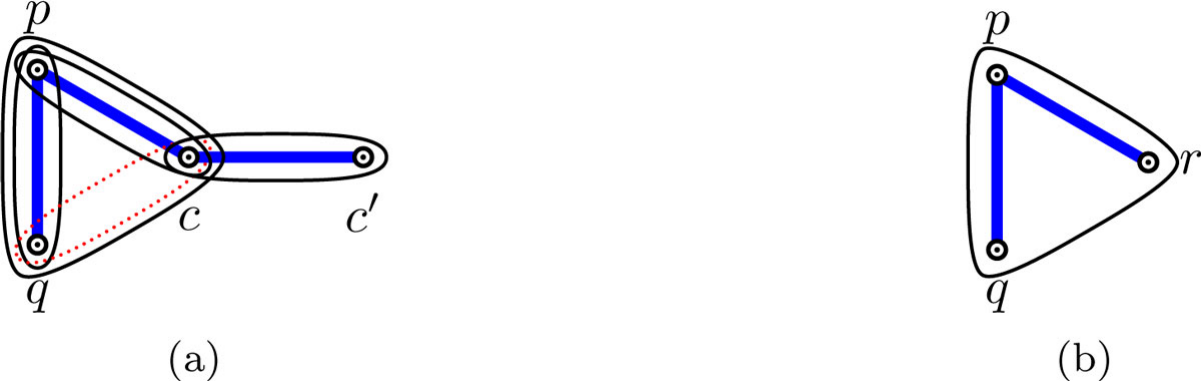
**A covering of the (a) 2-clause gadget; and (b) 3-clause gadget, where literal *p *is satisfied**.

For a 2-clause (resp., 3-clause) *c *containing literals *p*, *q *(resp., and also *r *for a 3-clause), without loss of generality let *p *be a literal that is satisfied in *c *(there has to be such a literal since it is a satisfying assignment). If *c *is a 2-clause (resp., 3-clause), we cover the corresponding gadget as depicted in Figure 4a (resp., 4b).

In the above covering, since exactly *m*_2 _+ *n *hyperedges were removed, and since it is easy to verify that each vertex has degree at most 2, it follows that it is a valid covering of $H_{\phi }$.

"⇐" Now we show that if $H_{\phi }$ has a valid covering then ϕ  is satisfiable.

For hypergraph $H_{\phi }=\left(V,E\right)$, we say that a graph *G *= (*V*, *E*') *selects *a literal vertex for *x *∈ *X *of $H_{\phi }$ if *x *is adjacent to two edges of *G *in some clause gadget of $H_{\phi }$. Obviously, selected vertices of *G *correspond to a satisfying truth assignment of ϕ  if and only if

(i) in every clause gadget, at least one literal vertex is selected, and

(ii) for every *x *∈ *X*, at most one of *x *and ¬*x *is selected.

We call a graph *G *= (*V*, *E*') an *expected behavior *covering of $H_{\phi }=\left(V,E\right)$ when each variable (resp., clause) gadget of $H_{\phi }$ is covered in a way depicted in Figure 3 (resp., 4). It is easy to verify the following observation.

**Observation 1 ***If a valid covering G *= (*V, E*') *of *$H_{\phi }=\left(V,E\right)$*is also an expected behavior covering of *$H_{\phi }$, *then G corresponds to a satisfying truth assignment of ϕ*.

In the remainder of this lemma, we will give a set of transformations that converts a valid covering into an expected behavior covering while preserving the validity of the covering at each step. Assume that we have a valid covering of *H_ϕ_*.

We say that a variable gadget is *undecided *in a valid covering of *H_ϕ _*if neither of its two main hyperedges is removed. We first show that we can assume that there are no undecided variable gadgets.

**Claim 1 ***We can transform a valid covering of H_ϕ _into a valid covering that contains no undecided variable gadgets*.

*Proof*. To prove this claim we do a case analysis on the possible configurations that an undecided variable gadget can have in a valid covering of *H_ϕ_*, and show how we can locally transform each one to a decided configuration without affecting the validity of the covering of *H_ϕ_*.

**Figure 5:**
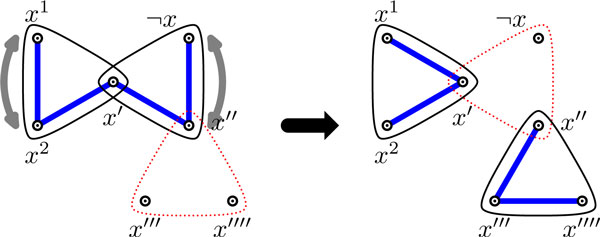
**The transformation in the case when the auxiliary hyperedge of the variable gadget is removed**.

**Figure 6:**
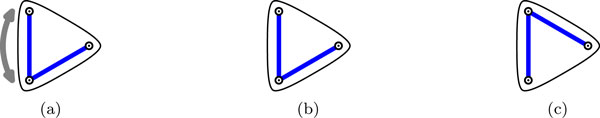
**(a) a 3-edge with a double arrow pointing to two vertices**. (b)-(c) the two configurations that are represented by (a).

First, assume that the auxiliary hyperedge is removed in an undecided variable gadget. The set of possible configurations that the gadget can be in is depicted on the left in Figure 5. In this figure (as in all remaining figures) double-headed arrows pointing to two vertices in a 3-edge represent the two coverings of this 3-edge as explained in Figure 6.

We can transform any configuration of Figure 5 to the decided configuration on the right. It is easy to see that in the transformed configuration, the number of hyperedges removed is the same as in any initial configuration, and that vertices *x*', *x*'', *x*''' and *x*'''' (which do not intersect any vertex outside this variable gadget) have degree at most 2. Finally, since each initial configuration of Figure 5 is part of a valid covering of *H_ϕ_*, and the degree of any literal vertex (*x*^1^, *x*^2 ^and ¬x) affected by the transformation has only decreased or remained the same, it follows that the covering of *H_ϕ _*remains valid after this local transformation.

**Figure 7:**
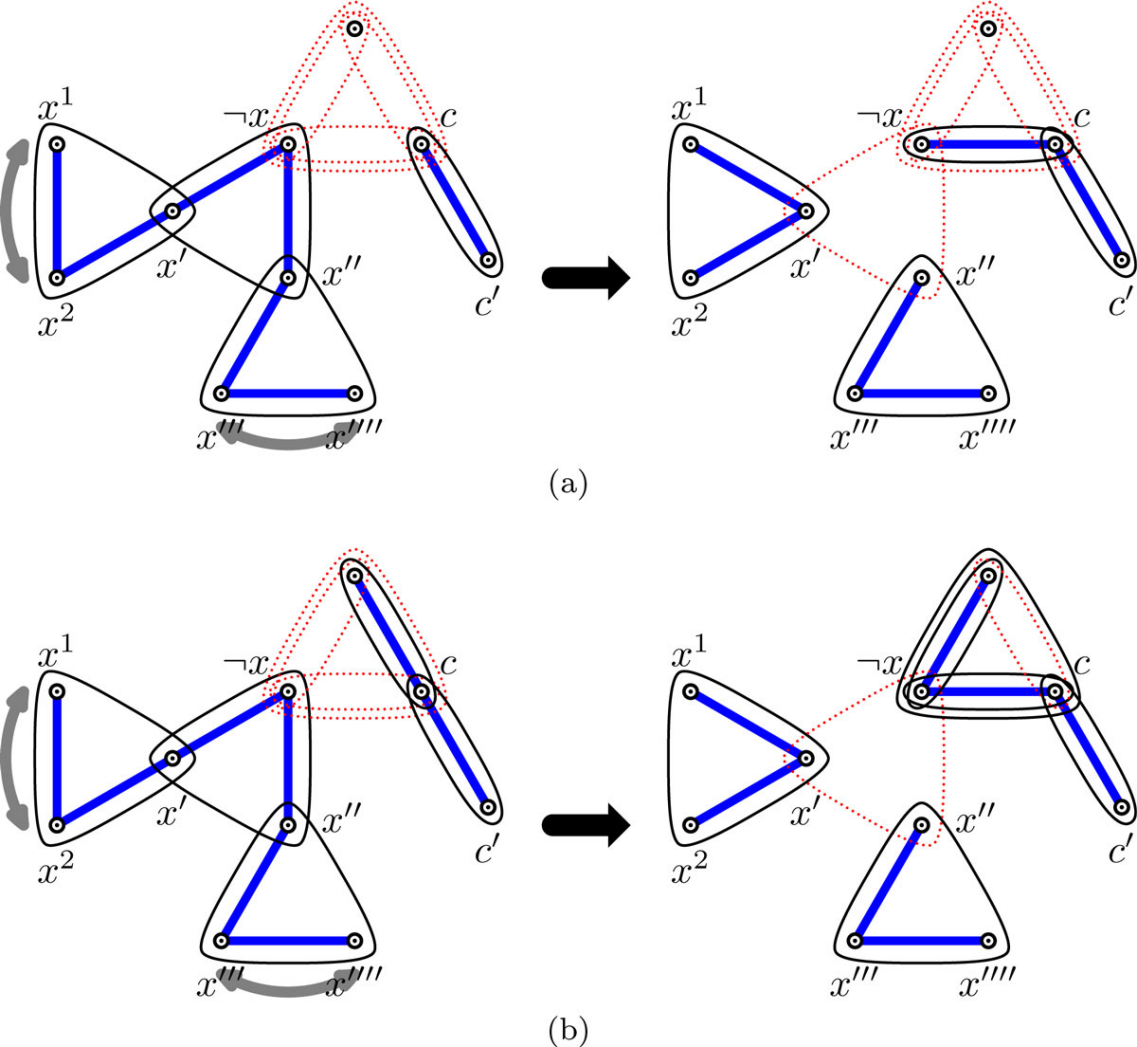
**Two sets of possible configurations of an undecided variable gadget and the corresponding transformation of the covering**. (Note that if edge {*c*, *c*'} is also missing in either initial configuration on the left, that the corresponding configuration on the right still applies, since *c *and *c*' do not intersect any vertex outside this variable gadget, and the number of hyperedges removed has not increased.)

Hence, we can assume that the auxiliary hyperedge is present in any undecided variable gadget. Without loss of generality we can then assume that any configuration of the undecided variable gadget must be in one of the two forms depicted on the left in Figure 7. In each case, we can perform the corresponding transformation shown in Figure 7. Again it is easy to see that the number of hyperedges removed has not increased, that vertices *x*', *x*'', *x*''', *x*'''', *c *and *c*' (which do not intersect any vertex outside of what is shown here) have degree at most 2, and that degree of any involved literal vertex has not increased. Hence, the covering remains valid. QED

We have the following claim.

**Claim 2 ***In any valid covering of H_ϕ_*, *at least one hyperedge is removed from each 2-clause gadget*.

*Proof*. If no hyperedge is removed from the 2-clause gadget (*i.e.*, all hyperedges are covered) in a valid covering of *H_ϕ_*, then vertex *c *(see Figure 2b) has degree 3, which contradicts the fact that this 2-clause gadget is part of a valid covering of *H_ϕ_*. QED

By Claims 1 and 2, at least *n *+ *m*_2 _hyperedges have been removed from the variable and 2-clause gadgets, and since in any valid covering this is the maximum number of hyperedges which can be removed, we have the following corollary.

**Corollary 1 ***We can transform a valid covering of H_ϕ _into a valid covering where:*

*(a) exactly one hyperedge is removed from each variable gadget and each 2-clause gadget of H_ϕ_*, *and*

*(b) no hyperedge is removed from any 3-clause gadget*.

We have the following claim.

**Claim 3 ***We can transform a valid covering of H_ϕ _into an expected behavior valid covering*.

**Figure 8:**
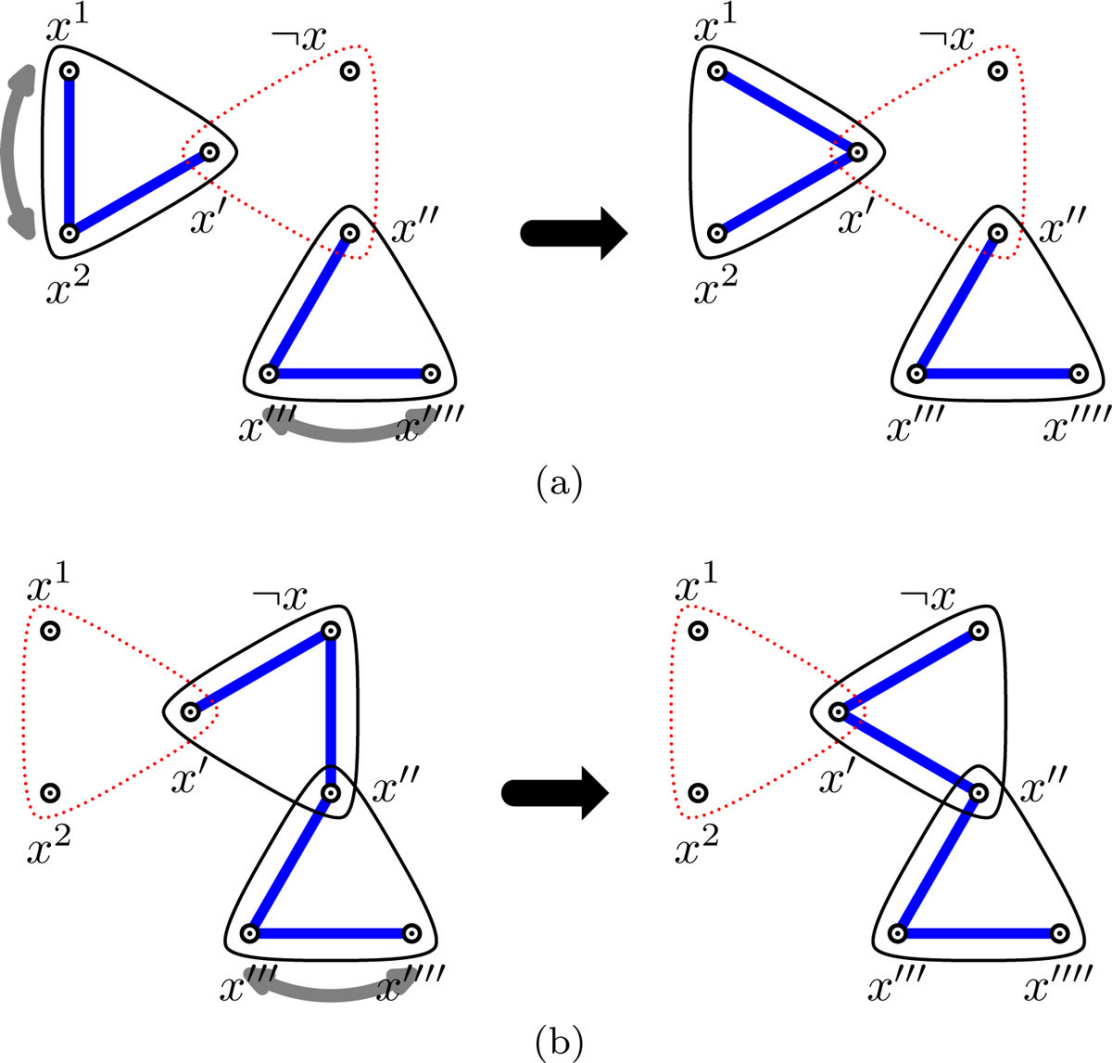
**Two sets of possible configurations of a decided variable gadget without expected behavior and the corresponding transformations**.

*Proof*. Firstly, in the valid covering of *H_ϕ _*we can assume, by Claim 1 and Corollary 1, that exactly one main hyperedge is removed and the auxiliary hyperedge is not removed from each variable gadget. However, this does not imply expected behavior. All possible configurations of a decided variable gadget without expected behavior and their corresponding transformations to the expected behavior covering are shown in Figure 8. Analogous to the proof of Claim 1, these local transformations do not affect validity of the covering.

**Figure 9:**
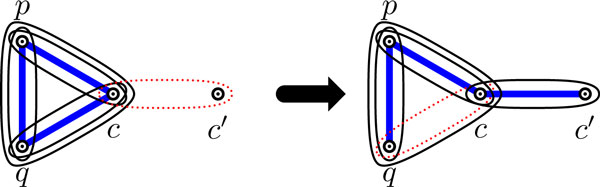
**The only possible configuration of a 2-clause gadget without expected behavior and the corresponding transformation**.

Secondly, in the valid covering of *H_ϕ _*we can assume, by Corollary 1, that exactly one hyperedge is removed from each 2-clause gadget. Assume now that a 2-clause gadget is not expected behavior covered. The only possible such configuration can be transformed to the expected behavior covering as shown in Figure 9. Again, these local transformations do not affect validity of the covering.

Thirdly, in the valid covering of *H_ϕ _*we can assume, again by Corollary 1, that the 3-clause gadget is covered, and hence it is also expected behavior covered (see Figure 4b). Since all gadgets are expected behavior covered in this valid covering of *H_ϕ_*, the claim holds. QED

It follows by Observation 1 and Claim 3, that if *H_ϕ _*has a valid covering, then *ϕ *is satisfiable. This completes the proof of the lemma. QED

Finally, since the construction of *H_ϕ _*is polynomial, then by Lemma 2 it follows that the 23UCR Problem is NP-complete. QED

Let the component-Ci1P by Row Removal Problem be the corresponding decision version of the MAX-ROW-component-Ci1P Problem as follows.

**Problem 3 (The component-Ci1P by Row Removal Problem) ***Given a binary matrix M and an integer k, can we obtain a submatrix that is component-mCi1P by removing at most k rows from M?*

We now show that the component-Ci1P by Row Removal Problem is NP-complete for matrices with rows of degrees 2 and 3.

The following lemma shows the correspondence between the component-Ci1P by Row Removal Problem for matrices with rows of degrees 2 and 3 and the 23UCR Problem. A 2,3-uniform hypergraph *H *= (*V*, *E*) can be represented by a binary matrix *B_H _*with |*V*| columns and |*E*| rows, where for each hyperedge *h *∈ *E*, we add a row with 1's in the columns corresponding to the vertices in *h *and 0's everywhere else. Obviously, there is a one-to-one correspondence between 2,3-uniform hypergraphs and such matrices.

**Lemma 3 ***A 2,3-uniform hypergraph H = *(*V, E*) *can be covered by a collection of disjoint cycles and paths after removing at most k hyperedges from E if and only if matrix B_H _has the component-Ci1P after removing at most k rows*.

*Proof*. Assume first that *H *has a covering *G *that consists of a collection of disjoint cycles and paths after removing at most *k *hyperedges from *E*. Remove the (at most *k*) rows from *B_H _*that correspond to the hyperedges removed from *E*. Each path (resp., cycle) *O *of *G *defines a cyclic order on its set of vertices. Consider the cyclic ordering of the columns of each component of *B_H _*corresponding to *O*. It is easy to see that each such cyclic ordering is a Ci1P ordering of its corresponding component, and hence *B_H _*has the component-Ci1P after removing at most *k *rows.

Conversely, assume that each component *C *= {*v*_1_,...,*v*_|*C*|_} of the submatrix of *B_H _*obtained by removing at most *k *rows is Ci1P with respect to cyclic order $\pi =v_{i_{1}},\ldots ,\;v_{i{\;}_{\left|\;C\;\right|}}$ of its columns. Consider the following covering *G *of *H*, after removing the (at most *k*) hyperedges from *E *that correspond to the rows removed from *B_H_*: for every hyperedge, pick the edge between two adjacent columns/vertices in *π*. Note that every picked edge is $\left\{v_{i_{j}},v_{i_{j+1}}\right\}$ for some *j*, or $\left\{v_{i_{\left|C\right|}},v_{i_{1}}\right\}$. Hence, *G *consists of a collection of disjoint cycles and paths. QED

By Theorem 2 and Lemma 3 it follows that the component-Ci1P by Row Removal Problem is NP-complete for matrices with rows of degrees 2 and 3. Since this decision problem is NP-complete, it follows that the MAX-ROW-component-Ci1P Problem is also NP-complete for matrices with rows of degrees 2 and 3.

**Theorem 3 ***The MAX-ROW-component-Ci1P Problem is NP-complete*.

## Discussion/Conclusion

There are exact optimization [12,19,20] or empirical [13-15] fast methods to construct ancestral adjacencies which do not necessarily form a linear signal. But to date, all linearization methods were heuristics or calls to Traveling Salesman Problem solvers [12-14,19]. Moreover, no method is currently adapted to reconstruct bacterial ancestral genome with plasmid(s), while this situation is common in the living world.

We report here two results: (1) a polynomial variant of the Linearization Problem, when the output allows paths and cycles and a maximum number of copies per gene, in the case of degree 2 matrices with weighted rows; and (2) an NP-completeness proof of the same problem for matrices with rows of degrees 2 and 3, even when multiplicities and weights are equal to one.

It is not the first time that a slight change in the formulation of a problem dramatically changes its computational status [10]. Even if such a relaxation is less realistic in certain contexts, solving the relaxation can also help to approach efficiently the constrained problem, like for DCJ and inversions/translocations for example [28,29]. More generally, 2-factors (spanning subgraphs composed of collections of vertex-disjoint cycles) have been used to approximate Traveling Salesman solutions [24], so genomes composed of several circular chromosomes can be a way to approximate solutions for linear ones.

Moreover, considering genomes composed of linear and circular segments is appropriate for bacterial genomes where linear segments can be seen as segments of not totally recovered circular chromosomes. Currently no ancestral genome reconstruction method is able to handle bacterial genomes with plasmids, but rather they are restricted to eukaryotes or bacterial chromosomes with a single circular chromosome. For example, Darling *et al. *[30] reconstruct the ancestral genomes of *Yersinia pestis *strains but are limited to the main chromosome by their method, while there are 3 plasmids in most current species, and they are of capital importance since they are suspected to have provoked the pathogenicity of the plague agent. So it is crucial to include them in evolutionary studies, which justifies our model for future biological studies.

Furthermore, genes are often duplicated in genomes, and in the absence of a precise and efficient phylogenetic context, which is still absent for bacteria (no ancestral genome reconstruction method is able to handle horizontal transfers for example), a multi-copy family translates into a multiplicity in the problem statements.

The ability to obtain such genomes in polynomial time from adjacencies also opens interesting perspectives for phylogenetic scaffolding of extant bacterial genomes [31] or more generally bacterial communities [32].

These applications are left as a future work.

## Competing interests

The authors declare that they have no competing interests.

## Authors' contributions

JM, MP, RW, CC and ET formalized and solved the linearization problems and wrote the paper.
